# Development and validation of an ultrasound‑based radiomics nomogram to predict lymph node status in patients with high-grade serous ovarian cancer: a retrospective analysis

**DOI:** 10.1186/s13048-024-01375-7

**Published:** 2024-02-22

**Authors:** Yue Qi, Jinchi Liu, Xinyue Wang, Yuqing Zhang, Zhixun Li, Xinyu Qi, Ying Huang

**Affiliations:** 1 Department of Ultrasound, Shengjing Hospital of China Medical University, No. 36 Sanhao Street, Heping District, Shenyang, Liaoning Province 110004 China; 2 Department of Oncology, Shengjing Hospital of China Medical University, Shenyang, Liaoning Province China

**Keywords:** High-grade serous ovarian cancer, Ultrasonography, Lymph nodes, Radiomics, Metastasis

## Abstract

**Background:**

Despite advances in medical imaging technology, the accurate preoperative prediction of lymph node status remains challenging in ovarian cancer. This retrospective study aimed to investigate the feasibility of using ultrasound-based radiomics combined with preoperative clinical characteristics to predict lymph node metastasis (LNM) in patients with high-grade serous ovarian cancer (HGSOC).

**Results:**

Patients with 401 HGSOC lesions from two institutions were enrolled: institution 1 for the training cohort (*n* = 322) and institution 2 for the external test cohort (*n* = 79). Radiomics features were extracted from the three preoperative ultrasound images of each lesion. During feature selection, primary screening was first performed using the sample variance F-value, followed by recursive feature elimination (RFE) to filter out the 12 most significant features for predicting LNM. The radscore derived from these 12 radiomic features and three clinical characteristics were used to construct a combined model and nomogram to predict LNM, and subsequent 10-fold cross-validation was performed. In the test phase, the three models were tested with external test cohort. The radiomics model had an area under the curve (AUC) of 0.899 (95% confidence interval [CI]: 0.864–0.933) in the training cohort and 0.855 (95%CI: 0.774–0.935) in the test cohort. The combined model showed good calibration and discrimination in the training cohort (AUC = 0.930) and test cohort (AUC = 0.881), which were superior to those of the radiomic and clinical models alone.

**Conclusions:**

The nomogram consisting of the radscore and preoperative clinical characteristics showed good diagnostic performance in predicting LNM in patients with HGSOC. It may be used as a noninvasive method for assessing the lymph node status in these patients.

**Supplementary Information:**

The online version contains supplementary material available at 10.1186/s13048-024-01375-7.

## Background

Ovarian cancer (OC) has the highest mortality rate among all gynecological malignancies [1]. Epithelial ovarian cancer (EOC) accounts for more than 95% of all OC cases [2]. Although considerable progress has been made in the diagnosis and treatment of EOC, its prognosis remains poor [3]. High-grade serous ovarian cancer (HGSOC) accounts for approximately 60% of EOC cases [4]. Most patients with HGSOC have advanced disease at the time of diagnosis, and their long-term survival rates are low [5, 6]. The International Federation of Gynecology and Obstetrics (FIGO) ovarian cancer staging system [7] does not include substaging of lymph nodes, except as a distant disease manifestation. However, it has been shown that lymph node metastasis (LNM) represents tumor infiltration and spread, the incidence of LNM is lower in early- than in late-stage disease. Lymph node status significantly affects the survival of patients with OC. Patients with LNM are usually classified as stage III or IV and have a poorer prognosis [8, 9].

Currently, surgical and histopathological diagnosis is the gold standard for staging of EOC. According to the National Comprehensive Cancer Network guidelines [10], the resection of enlarged or suspicious lymph nodes on preoperative imaging or intraoperative exploration is recommended. However, there is significant controversy regarding the use of lymph node dissection for staging OC. Several studies [9, 11] have demonstrated that systematic lymph node dissection does not provide any benefit, with no difference in progression-free survival or overall survival, and is associated with a higher incidence of complications.

Despite advances in medical imaging technology, the accurate preoperative prediction of lymph node status remains difficult. Computed Tomography (CT) with intravenous contrast is the first-line imaging method for staging and follow-up of OC according to the American College of Radiology guidelines [12]. However, according to a meta-analysis, the sensitivity of CT for predicting LNM is not ideal, only 0.47 [13, 14]. The diagnostic efficacy of magnetic resonance imaging (MRI) [15] and positron emission tomography/computed tomography (PET/CT) [16] is also not high. The overlap between reactive hyperplastic and metastatic lymph nodes is the most common reason for false positives and false negatives [17]. Therefore, it is necessary to explore methods for the preoperative prediction of LNM.

The essence of radiomics is to extract unrecognizable features from medical images and establish a relationship between these high-throughput features and a low-noise state [18, 19]. Currently, CT- and MRI-based radiomics have been applied for the individualized treatment of HGSOC [6, 20–22]. Researchers are attempting to establish radiomic models based on ultrasonography [23]. To the best of our knowledge, no previous studies have explored ultrasound-based radiomics to predict LNM in patients with HGSOC to date. Therefore, the aim of this study was to explore the feasibility of predicting the lymph node status using preoperative ultrasound imaging-based radiomics in patients with HGSOC as well as to investigate whether preoperative clinical parameters can assist in predicting LNM.

## Methods

### Patients

**Fig. 1:**
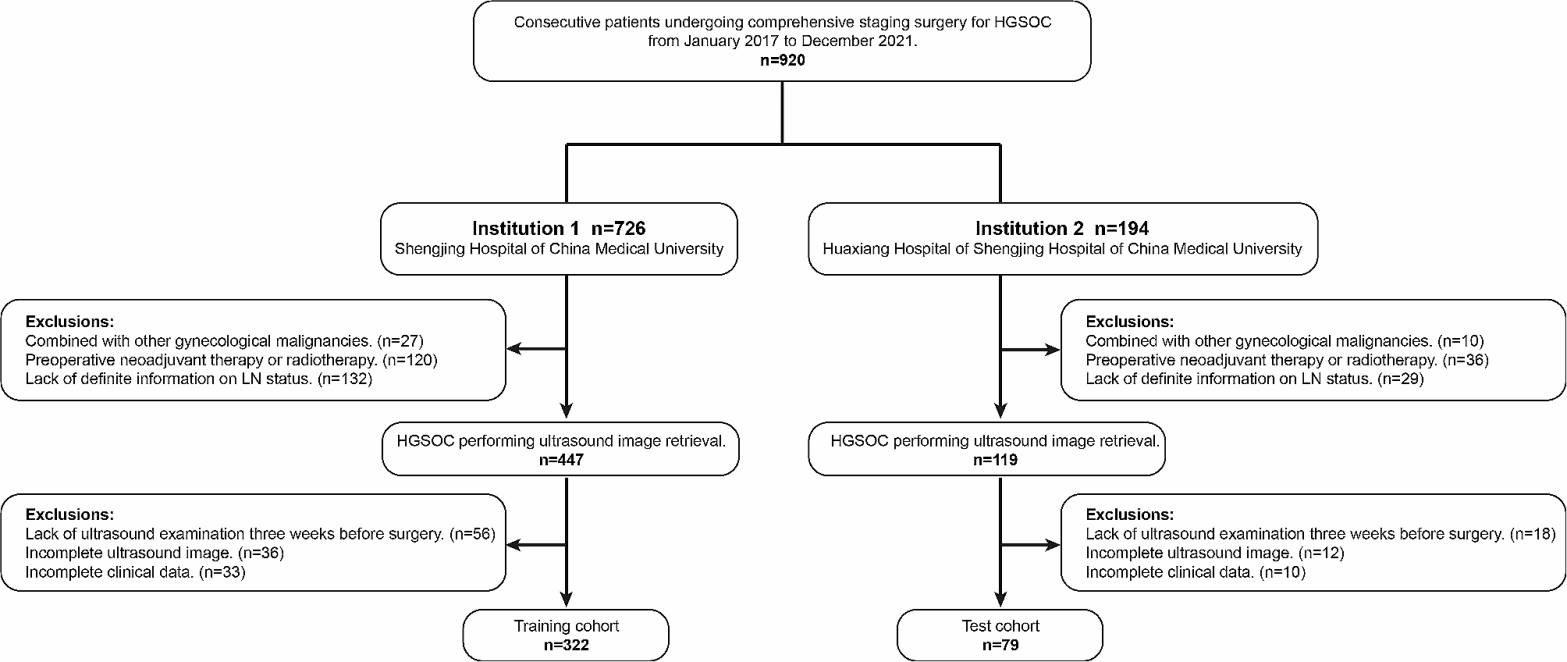
Flowchart of the inclusion and exclusion criteria for patients

We retrospectively reviewed 920 consecutive patients with HGSOC in two institutions (Institution 1: Shengjing Hospital of China Medical University; Institution 2: Huaxiang Hospital of Shengjing Hospital of China Medical University) from January 2017 to December 2021. All patients underwent comprehensive staging surgery with pelvic and para-aortic lymph node dissection. The inclusion criteria were as follows: (1) HGSOC diagnosed by postoperative pathology, (2) primary ovarian cancer, (3) ultrasound examination performed in our hospital within 3 weeks before surgery, (4) initial surgery, and (5) clear postoperative lymph node metastasis status. The exclusion criteria were as follows: (1) combination of other gynecological malignancies, (2) metastatic ovarian cancer, (3) preoperative adjuvant chemotherapy or radiotherapy, (4) unsatisfactory ultrasound images, and (5) incomplete clinical data. The endpoint event in this study was lymph node status determined by histopathologic findings after comprehensive staging surgery. Finally, our study included 401 eligible patients, with patients from institution 1 included in the training cohort (*n* = 322) and patients from institution 2 included in the test cohort (*n* = 79). A flowchart of the study is shown in Fig. 1.

### Tumor segmentation and feature extraction

Preoperative ultrasound images of all patients with HGSOC were retrieved using an picture archiving and communication system (PACS). Images from the final preoperative ultrasound examination were selected. For HGSOC with bilateral progression, larger and more complex solid lesions were selected for analysis. Three standard images were selected for each lesion: the largest section, including the most complex lesion component; the section orthogonal to the largest section; and the color Doppler imaging section of the solid component of the lesion. All lesions were manually delineated by a radiologist with three years of experience in gynecologic imaging using the Darwin research platform (https://arxiv.org/abs/2009.00908). All segments were confirmed by a senior radiologist with over 25 years of gynecological imaging experience, who was blinded to the pathological results corresponding to the images. If there was a difference between the two radiologists, the final region of interest (ROI) was confirmed through a discussion.

### Radiomic feature extraction and preprocessing

After determining the ROI, feature extraction was performed using the PyRadiomics package [24] built into the platform. A single ultrasound image provided 1125 features, and 3375 features were extracted from the three ultrasound images of each lesion. Seven categories were included: (1) shape2D statistics; (2) first-order statistics; (3) gray-level co-occurrence matrix (GLCM); (4) gray-level dependence matrix (GLDM); (5) gray-level run-length matrix (GLRLM); (6) gray-level size zone matrix (GLSZM); and (7) neighboring gray-tone difference matrix (NGTDM). A detailed description of these features is available at https://pyradiomics.readthedocs.io/en/latest/features.htm.

Data preprocessing is an important step in machine learning that can make the algorithm converge faster to obtain a more reasonable model. We used different ultrasound diagnostic instruments such as LOGIQ E9 (GE Co., NY, USA) and Mylab Class C (Esaote Co., Genoa, Italy) for ultrasound image acquisition. Therefore, before performing feature extraction, we normalized the original feature vector by subtracting the mean value from the extracted feature data and dividing it by the variance to minimize the differences caused by the different ultrasound instruments.

### Feature selection and radiomic model development

First, we used the optimal feature filter (i.e., sample variance F-value) to evaluate the linear correlation between each feature and the category label, and filtered the top 10% of the most relevant features with the largest F-value from the 3375 features. Subsequently, because some machine learning models can evaluate the importance of features, the classifier is trained iteratively until the classification performance is optimal by removing the features with the lowest importance at the end of each training session. We used a recursive feature elimination (RFE) method based on logistic regression (LR) to train the model iteratively with STEP set to 1. The features with the lowest weights were removed each time, and the top 12 features were selected. Models consisting of fewer than 12 features did not improve the classification performance.

After the optimal subset of features was derived from the above two feature selection steps, we used five supervised machine learning methods to build the classifier in the training cohort: support vector machine (SVM), K-nearest neighbor (KNN), random forest (RF), decision tree (DT), and LR. For SVM, the radial basis function (RBF) was chosen as the kernel function to fit the data. For RF and DT, overfitting was prevented by limiting the minimum sample size of the leaf nodes and the maximum tree depth. For LR, L1 regularization was used as a penalty. 10-fold cross-validation was performed for each classifier. The average area under the receiver operating characteristic (ROC) curve (AUC) and average sensitivity, specificity, and accuracy were provided as performance metrics for the cross-validation cohort. The classifier with the highest mean AUC was selected. Finally, the radscore for each patient was calculated according to a linear model based on LR and the radiomics model was constructed based on radscore.

### Establishment of the clinical and combined models

Radiomics can be used to extract high-dimensional features from images. However, owing to the heterogeneity of ultrasound images, some features closely related to the disease are equally relevant for predicting LNM, such as the lesion size, unilateral or bilateral involvement, presence of ultrasonography (US)-reported pelvic fluid, presence of US-reported peritoneal thickening, and presence of US-reported pelvic wall nodules. We recorded the above information from the US report and collected clinical data from the Hospital Information System (HIS), including age, menopausal status, and preoperative serological indicators (cancer antigen 125 (CA125) levels, human epididymal protein 4 (HE4) levels, carcinoembryonic antigen (CEA) levels, and cancer antigen 724 (CA724) levels). Lesion size, US-reported pelvic fluid, age, CA125, HE4, CEA, and CA724 were set as continuous variables. The other features were set as categorical variables.

We used the R language ‘mlr3’ package to construct a LR-based machine learning feature screener for clinical characteristics. A 10-fold cross-validation with 20 iterations was used to select the characteristics included in clinical model with the best AUC performance. A clinical model was developed using these clinical characteristics. To explore whether combining the radscore with the relevant clinical characteristics could further improve the predictive performance of the model, we combined the radscore and relevant clinical characteristics to build a multivariate logistic regression model and constructed a nomogram.

### External test and evaluation of the models

**Fig. 2:**
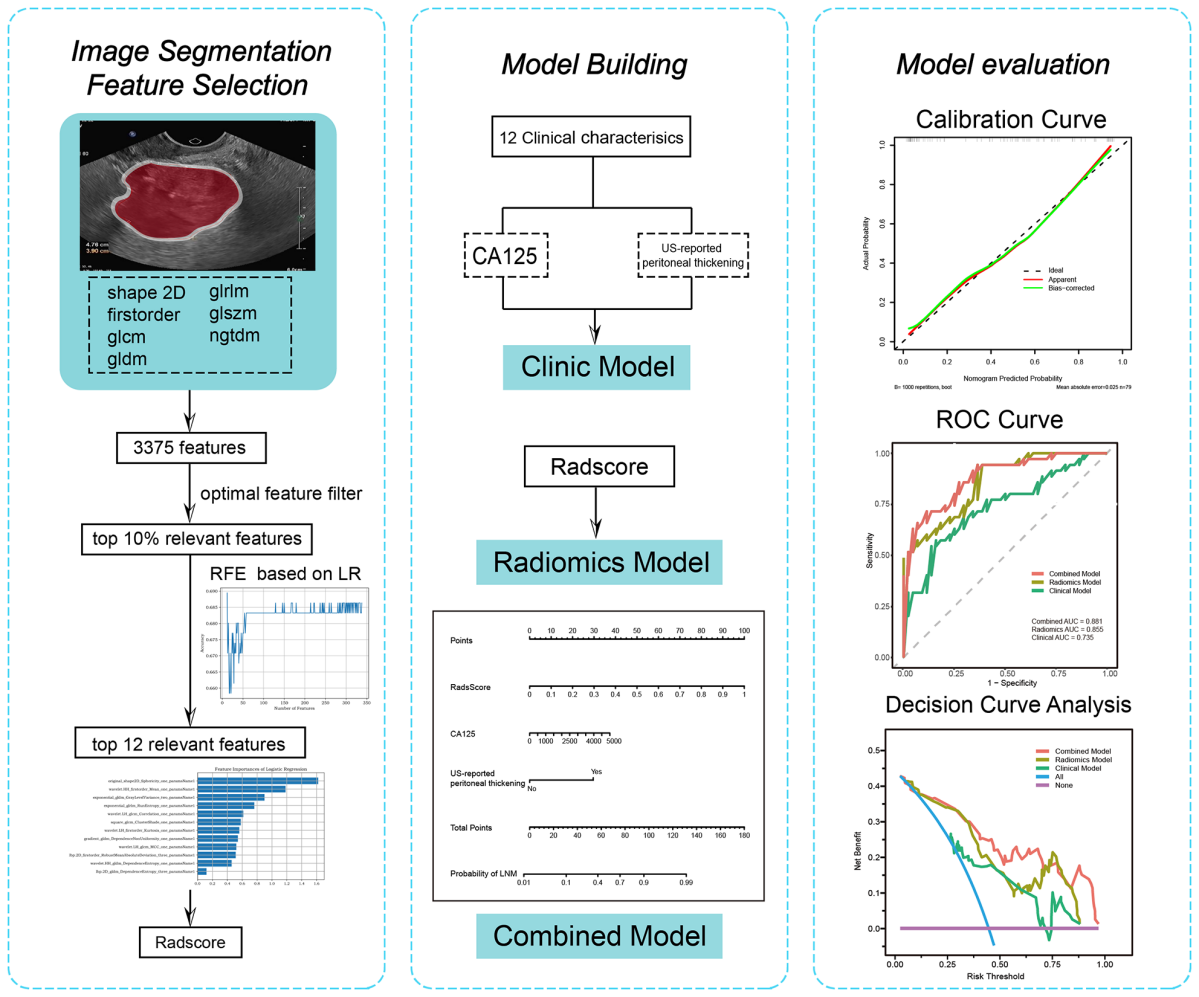
Workflow of this study

Three models were applied to the external test cohorts. Decision curve analysis (DCA) was performed to illustrate the net clinical benefits derived from the three models. Calibration curves were used to assess the nomogram performance. The overall workflow of the radiomics model development and validation is displayed in Fig. 2.

### Statistical analysis

All statistical analyses were performed using the R version 4.1.3 (R Foundation for Statistical Computing, Vienna, Austria. URL https://www.R-project.org/.). The statistical significance level was set at 0.05. The chi-square test was used to compare categorical variables, and the Mann-Whitney U test was used to compare continuous variables. The diagnostic efficiency of the models was evaluated using ROC curves and quantified using the AUC. The sensitivity, specificity, and accuracy were calculated to quantify various aspects of the models’ diagnostic ability.

## Results

### Patient characteristics

Among the 401 patients included in this study, the mean age was 54.6 ± 8.71 years, 173 (43.1%) had postoperative pathologically confirmed LNM, and 228 (56.9%) had postoperative pathologically confirmed no-LNM; 65.6% of the patients were in the postmenopausal state. The mean maximum diameter of the lesion was 9.46 cm. Suspicious peritoneal thickening and pelvic wall nodules were detected on US in 25.9% and 20.7% of patients, respectively. There were no statistical differences in the clinical characteristics of the patients between the training and test cohorts (*p* > 0.01). The clinical characteristics of patients in the training and test cohorts are presented in Table 1. In the training cohort, we found significant differences (*p* < 0.05) between the LNM-negative and LNM-positive groups with regard to US-reported pelvic fluid, laterality, US-reported peritoneal thickening, US-reported pelvic wall nodules, CA125, HE4, CEA, and CA724 levels, with the LNM-positive group having a deeper pelvic fluid depth, greater odds of peritoneal thickening and pelvic wall nodules, and significantly higher serological indicators than the LNM-negative group. In contrast, only CA125, HE4 levels and US-reported peritoneal thickening were significantly different between the two groups in the test cohort.

**Table 1:** Clinical characteristics of the patients in the training and test cohorts

Characteristics	Training Cohort (*n* = 322)			Test Cohort (*n* = 79)		
	LNM negative	LNM positive	p value	LNM negative	LNM positive	p value
	*n* = 184	*n* = 138		*n* = 44	*n* = 35	
RadsScore, mean (SD)	0.23 (0.21)	0.69 (0.27)	<0.05	0.34 (0.29)	0.74 (0.23)	<0.05
Age, mean (SD)	54.7 (8.40)	53.3 (9.09)	0.163	56.8 (7.93)	55.6 (9.34)	0.556
Menopausal status, n (%)			0.391			1
No	61 (33.2%)	53 (38.4%)		13 (29.5%)	11 (31.4%)	
Yes	123 (66.8%)	85 (61.6%)		31 (70.5%)	24 (68.6%)	
US-reported pelvic fluid, n (%)	2.16 (2.41)	3.91 (3.23)	<0.05	2.01 (1.92)	2.84 (2.87)	0.147
Lateral			<0.05			0.116
Unilateral	112 (60.9%)	63 (45.7%)		29 (65.9%)	16 (45.7%)	
Bilateral	72 (39.1%)	75 (54.3%)		15 (34.1%)	19 (54.3%)	
US-reported peritoneal thickening, n (%)			<0.05			<0.05
No	163 (88.6%)	78 (56.5%)		38 (86.4%)	18 (51.4%)	
Yes	21 (11.4%)	60 (43.5%)		6 (13.6%)	17 (48.6%)	
US-reported pelvic wall nodules, n (%)			<0.05			0.102
No	163 (88.6%)	95 (68.8%)		37 (84.1%)	23 (65.7%)	
Yes	21 (11.4%)	43 (31.2%)		7 (15.9%)	12 (34.3%)	
Maximum diameter, mean (SD)	9.50 (4.18)	9.55 (4.03)	0.912	9.15 (3.36)	9.24 (3.73)	0.91
CA125, median (IQR)	254 [77.9;564]	840 [398;1709]	<0.05	302 [74.6;613]	530 [139;1097]	<0.05
HE4, median (IQR)	191 [105;363]	448 [243;710]	<0.05	152 [102;229]	251 [152;518]	<0.05
CEA, median (IQR)	1.52 [0.94;2.08]	1.17 [0.78;1.98]	<0.05	1.39 [0.88;2.13]	1.52 [0.96;2.14]	0.487
CA724, median (IQR)	6.91 [2.83;20.8]	19.6 [7.15;64.2]	<0.05	7.95 [4.26;13.7]	10.7 [6.28;21.5]	0.186

*Abbreviations* LNM, lymph node metastasis; SD, standard deviation; IQR, interquartile range; CA125, cancer antigen 125; HE4, human epididymal protein 4; CEA, carcinoembryonic antigen; CA724, cancer antigen 724

### Feature selection and construction of the radiomic model

A total of 3375 features were extracted from 401 lesions, and 12 features that were highly correlated with LNM were selected using 2-step feature selection. To derive the optimal prediction model, we selected five machine learning algorithms for classifier construction in the training cohort and compared the performances of several classifiers using 10-fold cross-validation. The performance of the five classifiers is shown in Table 2. The LR model achieved better classification performance with a mean AUC, sensitivity, specificity, and accuracy of 0.876, 0.688, 0.860 and 0.789, respectively.

**Table 2:** Diagnostic efficiency of different classifiers in the training and test cohorts

Classifiers	Training cohort				10 fold cross validation			
	AUC (95% CI)	SEN	SPE	ACC	Mean AUC	Mean SEN	Mean SPE	Mean ACC
SVM	0.936 (0.906,0.967)	0.916	0.830	0.866	0.843	0.658	0.870	0.782
KNN	0.907 (0.878,0.937)	0.906	0.728	0.804	0.811	0.595	0.846	0.733
RF	0.988 (0.980,0.996)	0.978	0.951	0.963	0.823	0.627	0.871	0.767
DT	0.893 (0.858,0.928)	0.797	0.859	0.832	0.774	0.663	0.744	0.705
LR	0.899 (0.864,0.933)	0.877	0.804	0.835	0.876	0.688	0.860	0.789

*Abbreviations* SVM, support vector machine; KNN, K-nearest neighbor; RF, random forest; DT, decision tree; LR, logistic regression; AUC, area under the curve; CI, confidence interval; SEN, sensitivity; SPE, specificity; ACC, accuracy

We derived the radscore for each patient from these 12 features using a linear model based on LR and then applied the radscores to build a radiomic model. The scoring formula and the radscores for each patient are presented in Table S1. There was a significant difference in the radscore between patients with and without LNM in the training cohort (0.69 ± 0.27 vs. 0.23 ± 0.21; *p*<0.05) and test cohort (0.74 ± 0.23 vs. 0.34 ± 0.29; *p*<0.05). The AUC value of the radiomic model based on the radscore was 0.930 (95% CI: 0.902–0.958) in the training cohort and 0.881 (95% CI: 0.801–0.954) in the test cohort.

### Clinical model construction and evaluation

We filtered clinical characteristics using a LR-based machine learning feature filter. After 20 iterations of 10-fold cross-validation, CA125 and US-reported peritoneal thickening were identified as the variables for clinical model which AUC was 0.762. The AUC of clinical model for predicting LMN was 0.770 (95% CI: 0.719–0.822) in the training cohort and 0.735 (95% CI: 0.622–0.848) in the test cohort.

### Combined model construction and evaluation

**Fig. 3:**
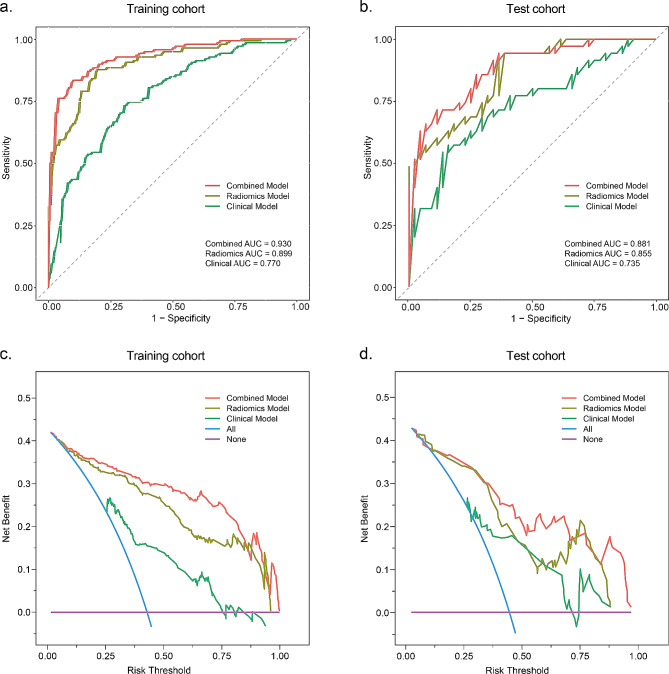
Predictive performance of the radiomic, clinical and combined models in the training and test cohorts. (**a**, **b**) show the ROC curves of the different models in the training and test cohorts. Decision curve analysis (**c**, **d**) illustrates the net clinical benefits of the prediction model. The y-axis represents the net benefit and x-axis represents the threshold probability. The blue line indicates “treat all” and the pink horizontal line denotes “treat none.” ROC, receiver operating characteristic curve

We performed LR using the independent clinical predictors and radscore and constructed a combined model. We compared the diagnostic performance of the radiomic, clinical, and combined models. Table 3; Fig. 3a, b show the sensitivity, specificity, accuracy, and AUC of the three models in the training and test cohorts. We observed that the AUC of the combined model improved from 0.899 (95%CI: 0.864–0.933) to 0.930 (95% CI: 0.902–0.958) in the training cohort and from 0.855 (95% CI: 0.774–0.935) to 0.881 (95% CI: 0.801–0.954) in the test cohort. DCA showed that the combined model had a higher overall net benefit at the threshold probability (Fig. 3c, d).

**Table 3:** Diagnostic efficiency of the clinical, radiomic, and combined models in the training and test cohorts

	Training cohort				Test cohort			
	AUC (95% CI)	SEN	SPE	ACC	AUC (95% CI)	SEN	SPE	ACC
Clinical model	0.770 (0.719–0.822)	0.732	0.696	0.711	0.735 (0.622–0.848)	0.571	0.841	0.722
Radiomics model	0.899 (0.864–0.933)	0.877	0.804	0.835	0.855 (0.774–0.935)	0.943	0.636	0.772
Combined model	0.930 (0.902–0.958)	0.833	0.902	0.873	0.881 (0.801–0.954)	0.714	0.886	0.810

*Abbreviations* AUC, area under the curve; CI, confidence interval; SEN, sensitivity; SPE, specificity; ACC, accuracy

**Fig. 4:**
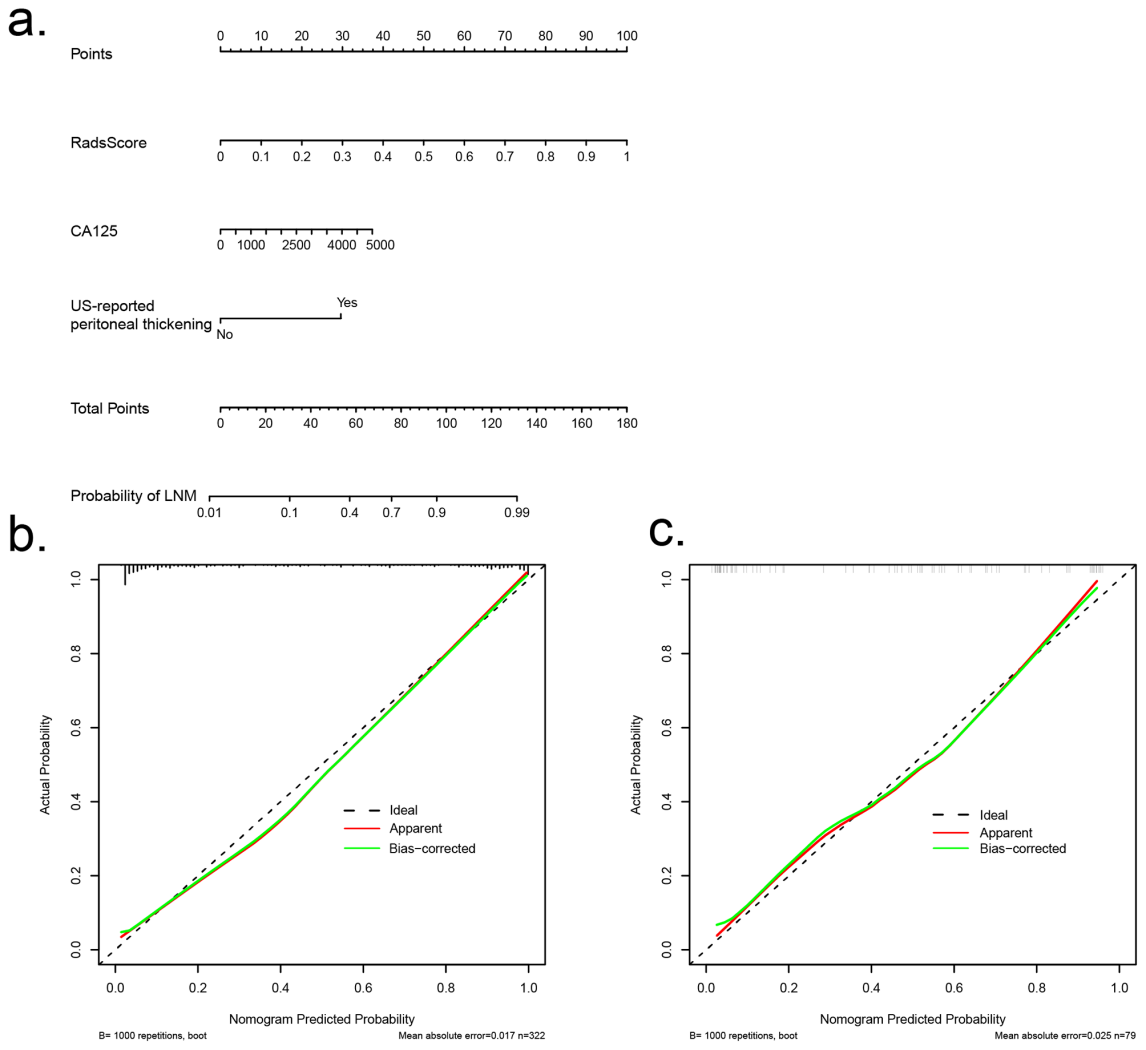
(**a**) Nomogram for predicting lymph node metastasis of HGSOC based on radscore and clinical characteristics. In the nomogram, a vertical line was first made according to the Radscore to determine the corresponding value of points. Similarly, the CA125 and US-reported peritoneal thickening values were also determined. The total points were the sum of the three points above. Finally, a vertical line was made according to the value of the total points to determine the probability of LNM. (**b**, **c**) show the calibration curves of the nomogram developed in the training and test cohorts. HGSOC, high-grade serous ovarian cancer

The combined model was then used to construct a nomogram (Fig. 4a). Calibration curves of the combined model are shown in Fig. 4b, c. The alignment of the dashed and solid lines indicates a good agreement between the predicted results of LMN and the true state in the training and test cohorts.

## Discussion

In our study, we constructed a prediction model to predict LNM in HGSOC. We used three preoperative ultrasound images of patients with OC to identify radiomic features and calculate radscore. A nomogram was created using radscore, serologic CA125 levels, and US reported peritoneal thickening. This nomogram can predict the probability of LNM in patients with ovarian cancer preoperatively. The model exhibited good performance and can help in making individualized preoperative decisions.

Since the development of radiomics, the relationship between the high-throughput information embedded in the images and the biological behavior of the disease has been the focus of research. We believe that the highly aggressive and metastatic tendencies during tumor development cause changes in the imaging presentation that are difficult to observe with the naked eye during the early stages. In previous studies, researchers tended to look for direct imaging signs of metastatic lymph nodes, such as an oval shape and disappearance of lymphatic portals, while ignoring the features of the tumor itself [13]. Many histological micrometastases may not be morphologically altered, whereas reactive hyperplastic lymph nodes may exhibit changes in size and morphology. Studies have shown that radiomics based on primary lesions can identify LNM in cervical cancer [23]. The prediction of LNM based on imaging features of the primary tumor is in an exploratory stage.

In this study, we extracted a large number of radiomic features from US images. During feature selection, primary screening was first performed using the sample variance F-value, followed by RFE to filter out the 12 most important and stable features for predicting LNM. RFE has been increasingly adopted as a feature selection method to obtain key combinations of variables that maximize the model performance by adding or removing specific feature variables [25]. We used radiomic features to construct different machine learning classifiers. The AUCs of the five classifiers in the test cohort ranged from 0.774 to 0.876. As a linear regression method, LR allows for the output of probabilities for binary classification problems. Therefore, we chose LR as the classifier to derive the radscore and proceeded to the next step of model construction.

Previously, radiomics based on CT and MRI have been used to predict metastasis in ovarian cancer [20, 21, 26]. Some researchers have used CT and PET to predict pelvic and/or para-aortic LNM in patients with advanced EOC, and the specificity of the obtained radiomic model for predicting high-risk lymph nodes was reportedly 78.3% [27]. However, the sample size of this study was small and the credibility of the conclusions is speculative. Yao et al. [28] developed a model for predicting the lymph node status based on PET images of patients with ovarian cancer using residual neural networks and SVM for modeling. Their model had an AUC of 0.92 in the test cohort, but the model only included patients with early-stage ovarian cancer. However, most patients are already in an advanced stage at diagnosis. The clinical stage of OC was not limited in our study, which may have greater clinical applicability.

In addition to preoperative imaging, serum tumor markers are measured in patients with suspected ovarian cancer. Serum CA-125 and HE4 levels are considered clinical predictors of survival and treatment response in patients with EOC, but there is no conclusive evidence on whether serum tumor markers are predictive of LNM, and they vary widely between different study populations [29, 30]. Zhou et al. [31] found that preoperative serum CA125 level > 740 U/mL was a risk factor for LNM in patients with EOC. It has also been suggested that CA125 levels are not associated with LNM in early OC [32]. Increased HE4 levels promote ovarian cancer cell invasion and metastasis through certain signaling pathways [33]. However, in our analysis, there may be a role for serum CA125 levels in predicting lymph node metastasis in OC, which is consistent with some studies. While HE4, CA724 and CEA levels were excluded in our clinical model. Previous studies have investigated the predictors of LNM in OC and concluded that high-grade serous tumors, positive peritoneal cytology, advanced clinical stage, interval surgery, and bilateral adnexal involvement can predict LNM in patients with OC [34, 35]. This is consistent with our conclusion that peritoneal thickening on US images correlates with LNM.

However, our study has some limitations. First, our study included only patients with HGSOC and did not include other pathological subtypes, which limited the extrapolability of the model. For most patients with suspected ovarian cancer, a puncture biopsy of the lesion is performed for better treatment planning; therefore, most physicians already know the pathological type before surgery. However, we will continue training the model so that it can be applied to all pathological types of OC. Second, since this was a single-center retrospective study, the sample size needs to be improved. Whether the model can be applied to other hospitals and physicians with different levels of seniority remains to be investigated. Third, unlike most CT- and MRI-based radiomic studies, not all US images were acquired using the same US instrument, and different instrument parameter settings may have led to feature heterogeneity. This is because the widespread prevalence of US makes it impossible to perform US examinations in all gynecological patients in large general hospitals using the same instrument model. In this regard, we normalized the US images to make the distribution of each dimension similar in order to speed up model convergence and improve the model accuracy.

## Conclusions

In conclusion, we successfully developed a radiomic model based on preoperative US images and clinical characteristics and established a nomogram that can predict LNM more accurately in patients with HGSOC. Using this model, clinicians can decide whether to perform extensive lymph node dissection in patients with HGSOC, thereby avoiding the adverse effects of unnecessary lymph node dissection. In the future, we will incorporate more pathological types of ovarian cancer, increase the sample size, perform external validation across multiple hospitals, perform prospective validation to test the model, and develop US-based radiomics for patients with all stages of ovarian cancer to improve the diagnosis and prognosis.

### Electronic supplementary material

Below is the link to the electronic supplementary material.

Supplementary Material 1

## Data Availability

The images used in this study are available from the corresponding author upon request. All data analyzed in this study are included in the published article and its supplementary information files.

## Abbreviations

OC: ovarian cancer
EOC: epithelial ovarian cancer
HGSOC: high-grade serous ovarian cancer
FIGO: International Federation of Gynecology and Obstetrics
LNM: lymph node metastasis
RFE: recursive feature elimination
ROC: receiver operating characteristic
DCA: decision curve analysis
HIS: hospital Information system
SVM: support vector machine
KNN: K-nearest neighbor
RF: random forest
DT: decision tree
LR: logistic regression
RBF: radial basis function
ROI: region of interest
CT: computed tomography
MRI: magnetic resonance imaging
PET/CT: positron emission tomography/computed tomography
AUC: area under the curve
US: ultrasonography
GLCM: gray-level co-occurrence matrix
GLDM: gray-level dependence matrix
GLRLM: gray-level run-length matrix
GLSZM: gray-level size zone matrix
NGTDM: neighboring gray-tone difference matrix
CA125: cancer antigen 125
HE4: human epididymal protein 4
CEA: carcinoembryonic antigen
CA724: cancer antigen 724

## References

[1] Webb PM, Jordan SJ. Epidemiology of epithelial ovarian cancer. Best practice & research Clinical obstetrics & gynaecology. 2017;41. 10.1016/j.bpobgyn.2016.08.006 27743768

[2] Lheureux S, Braunstein M, Oza AM (2019). Epithelial ovarian cancer: evolution of management in the era of precision medicine. CA Cancer J Clin.

[3] Moufarrij S, Dandapani M, Arthofer E, Gomez S, Srivastava A, Lopez-Acevedo M (2019). Epigenetic therapy for ovarian cancer: promise and progress. Clin Epigenetics.

[4] Kim J, Park EY, Kim O, Schilder JM, Coffey DM, Cho C-H et al. Cell origins of High-Grade Serous Ovarian Cancer. Cancers. 2018;10(11). 10.3390/cancers10110433 PMC6267333 30424539

[5] Torre LA, Trabert B, DeSantis CE, Miller KD, Samimi G, Runowicz CD (2018). Ovarian cancer statistics, 2018. CA Cancer J Clin.

[6] Signorelli M, Guerra L, Pirovano C, Crivellaro C, Fruscio R, Buda A (2013). Detection of nodal metastases by 18F-FDG PET/CT in apparent early stage ovarian cancer: a prospective study. Gynecol Oncol.

[7] Javadi S, Ganeshan DM, Qayyum A, Iyer RB, Bhosale P (2016). Ovarian Cancer, the revised FIGO Staging System, and the role of imaging. AJR Am J Roentgenol.

[8] Bachmann C, Bachmann R, Fend F, Wallwiener D (2016). Incidence and impact of Lymph Node metastases in Advanced Ovarian Cancer: implications for Surgical Treatment. J Cancer.

[9] Harter P, Sehouli J, Lorusso D, Reuss A, Vergote I, Marth C (2019). A Randomized Trial of Lymphadenectomy in patients with Advanced Ovarian neoplasms. N Engl J Med.

[10] Armstrong DK, Alvarez RD, Backes FJ, Bakkum-Gamez JN, Barroilhet L, Behbakht K (2022). NCCN Guidelines® insights: ovarian Cancer, Version 3.2022. J Natl Compr Canc Netw.

[11] du Bois A, Reuss A, Harter P, Pujade-Lauraine E, Ray-Coquard I, Pfisterer J (2010). Potential role of lymphadenectomy in advanced ovarian cancer: a combined exploratory analysis of three prospectively randomized phase III multicenter trials. J Clin Oncol.

[12] Kang SK, Reinhold C, Atri M, Benson CB, Bhosale PR, Jhingran A (2018). ACR appropriateness Criteria® Staging and Follow-Up of Ovarian Cancer. J Am Coll Radiology: JACR.

[13] Mimoun C, Rouzier R, Benifla JL, Fauconnier A, Huchon C. Preoperative CT or PET/CT to Assess Pelvic and Para-Aortic Lymph Node Status in Epithelial Ovarian Cancer? A Systematic Review and Meta-Analysis. Diagnostics (Basel, Switzerland). 2021;11(10). 10.3390/diagnostics11101748 PMC8534764 34679446

[14] Sahdev A (2016). CT in ovarian cancer staging: how to review and report with emphasis on abdominal and pelvic disease for surgical planning. Cancer Imaging.

[15] Buys SS, Partridge E, Black A, Johnson CC, Lamerato L, Isaacs C (2011). Effect of screening on ovarian cancer mortality: the prostate, lung, colorectal and ovarian (PLCO) Cancer Screening Randomized Controlled Trial. JAMA.

[16] Tardieu A, Ouldamer L, Margueritte F, Rossard L, Lacorre A, Bourdel N et al. Assessment of Lymph Node involvement with PET-CT in Advanced Epithelial Ovarian Cancer. A FRANCOGYN Group Study. J Clin Med. 2021;10(4). 10.3390/jcm10040602 PMC7915394 33562725

[17] Eisenhauer EA, Therasse P, Bogaerts J, Schwartz LH, Sargent D, Ford R et al. New response evaluation criteria in solid tumours: revised RECIST guideline (version 1.1). European Journal of Cancer (Oxford, England: 1990). 2009;45(2):228 – 47. 10.1016/j.ejca.2008.10.026 19097774

[18] Lambin P, Rios-Velazquez E, Leijenaar R, Carvalho S, van Stiphout RGPM, Granton P et al. Radiomics: extracting more information from medical images using advanced feature analysis. European Journal of Cancer (Oxford, England: 1990). 2012;48(4):441-6. 10.1016/j.ejca.2011.11.036 PMC4533986 22257792

[19] Liu Z, Wang S, Dong D, Wei J, Fang C, Zhou X (2019). The applications of Radiomics in Precision diagnosis and treatment of Oncology: opportunities and challenges. Theranostics.

[20] Chen HZ, Wang XR, Zhao FM, Chen XJ, Li XS, Ning G (2021). The Development and Validation of a CT-Based Radiomics Nomogram to Preoperatively Predict Lymph Node Metastasis in High-Grade Serous Ovarian Cancer. Front Oncol.

[21] Song X-L, Ren J-L, Yao T-Y, Zhao D, Niu J (2021). Radiomics based on multisequence magnetic resonance imaging for the preoperative prediction of peritoneal metastasis in ovarian cancer. Eur Radiol.

[22] Li H, Cai S, Deng L, Xiao Z, Guo Q, Qiang J et al. Prediction of platinum resistance for advanced high-grade serous ovarian carcinoma using MRI-based radiomics nomogram. Eur Radiol. 2023. 10.1007/s00330-023-09552-w 36995415

[23] Jin X, Ai Y, Zhang J, Zhu H, Jin J, Teng Y (2020). Noninvasive prediction of lymph node status for patients with early-stage cervical cancer based on radiomics features from ultrasound images. Eur Radiol.

[24] van Griethuysen JJM, Fedorov A, Parmar C, Hosny A, Aucoin N, Narayan V (2017). Computational Radiomics System to Decode the Radiographic phenotype. Cancer Res.

[25] Sanz H, Valim C, Vegas E, Oller JM, Reverter F (2018). SVM-RFE: selection and visualization of the most relevant features through non-linear kernels. BMC Bioinformatics.

[26] Ai Y, Zhang J, Jin J, Zhang J, Zhu H, Jin X (2021). Preoperative prediction of Metastasis for Ovarian Cancer based on computed tomography Radiomics features and clinical factors. Front Oncol.

[27] Crestani A, Huchon C, Mezzadri M, Marchand E, Place V, Cornelis F (2022). A pre-operative radiological score to predict lymph node metastasis in advanced epithelial ovarian cancer. J Gynecol Obstet Hum Reprod.

[28] Yao H, Zhang X (2022). Prediction model of Residual Neural Network for Pathological Confirmed Lymph Node Metastasis of Ovarian Cancer. Biomed Res Int.

[29] Kim HS, Park NH, Chung HH, Kim JW, Song YS, Kang SB (2008). Significance of preoperative serum CA-125 levels in the prediction of lymph node metastasis in epithelial ovarian cancer. Acta Obstet Gynecol Scand.

[30] Kim HS, Kim JW, Cho JY, Chung HH, Park NH, Song YS (2009). The role of serum CA-125 levels in early-stage epithelial ovarian cancer on preoperative CT and MRI. Eur J Surg Oncology: J Eur Soc Surg Oncol Br Association Surg Oncol.

[31] Zhou J, Sun J-Y, Wu S-G, Wang X, He Z-Y, Chen Q-H (2016). Risk factors for lymph node metastasis in ovarian cancer: implications for systematic lymphadenectomy. Int J Surg (London England).

[32] Ditto A, Martinelli F, Reato C, Kusamura S, Solima E, Fontanelli R (2012). Systematic para-aortic and pelvic lymphadenectomy in early stage epithelial ovarian cancer: a prospective study. Ann Surg Oncol.

[33] Zhuang H, Tan M, Liu J, Hu Z, Liu D, Gao J (2014). Human epididymis protein 4 in association with annexin II promotes invasion and metastasis of ovarian cancer cells. Mol Cancer.

[34] Atallah D, Arab W, Dagher B, Khalil N, El Rawadi E, Atallah B (2021). Predictive factors of lymph node metastasis and pattern of repartition in patients with epithelial ovarian cancer. Future Oncol (London England).

[35] Bogani G, Tagliabue E, Ditto A, Signorelli M, Martinelli F, Casarin J (2017). Assessing the risk of pelvic and para-aortic nodal involvement in apparent early-stage ovarian cancer: a predictors- and nomogram-based analyses. Gynecol Oncol.

